# Computational Simulations Identify Pyrrolidine-2,3-Dione Derivatives as Novel Inhibitors of Cdk5/p25 Complex to Attenuate Alzheimer’s Pathology

**DOI:** 10.3390/jcm8050746

**Published:** 2019-05-24

**Authors:** Amir Zeb, Donghwan Kim, Sayed Ibrar Alam, Minky Son, Raj Kumar, Shailima Rampogu, Saravanan Parameswaran, Rahul Mahadev Shelake, Rabia Mukhtar Rana, Shraddha Parate, Jae-Yean Kim, Keun Woo Lee

**Affiliations:** 1 Division of Life Science, Division of Applied Life Science (BK21 Plus), Research Institute of Natural Science (RINS), Gyeongsang National University (GNU), 501 Jinju-daero, Jinju 52828, Korea; zebamir85@gmail.com (A.Z.); donghwanz@naver.com (D.K.); minky@gnu.ac.kr (M.S.); shailima.rampogu@gmail.com (S.R.); dr.p.saravanan.bi@gmail.com (S.P.); rabia.mukhtar.rana@gmail.com (R.M.R.); parateshraddha@gmail.com (S.P.); 2 Division of Life Sciences and Applied Life Science (BK 21plus), College of Natural Sciences, Gyeongsang National University (GNU), 501 Jinju-daero, Jinju 52828, Korea; ibrarchem@gmail.com; 3 Institute of Chemical Processes (ICP), Seoul National University, 1 Gwanak-ro, Gwanak-gu, Seoul 08826, Korea; rajkumar250287@gmail.com; 4 Division of Applied Life Sciences, Plant Molecular Biology and Biotechnology Research Center, Gyeongsang National University, Jinju 660-701, Korea; rahultnau@gmail.com

**Keywords:** Cdk5/p25 inhibition, molecular dynamics simulation, pharmacophore modeling, pyrrolidine-2,3-dione, inhibition of tau-protein phosphorylation, Alzheimer’s pathology

## Abstract

Mechanistically, neurotoxic insults provoke Ca^2+^-mediated calpain activation, which cleaves the cytoplasmic region of membrane-embedded p35 and produces its truncated form p25. Upon physical interaction, cyclin-dependent kinase 5 (Cdk5) and p25 forms hyperactivated Cdk5/p25 complex and causes severe neuropathological aberrations including hyperphosphorylated tau-mediated neurofibrillary tangles formation, Alzheimer’s symptoms, and neuronal death. Therefore, the inhibition of Cdk5/p25 complex may relieve p-tau-mediated Alzheimer’s pathology. Herein, computational simulations have identified pyrrolidine-2,3-dione derivatives as novel inhibitors of Cdk5/p25 complex. A ligand-based pharmacophore was designed and employed as 3D query to retrieve drug-like molecules from chemical databases. By molecular docking, drug-like molecules obtaining dock score > 67.67 (Goldcore of the reference compound) were identified. Molecular dynamics simulation and binding free energy calculation retrieved four pyrrolidine-2,3-dione derivatives as novel candidate inhibitors of Cdk5/p25. The root means square deviation of Cdk5/p25 in complex with candidate inhibitors obtained an average value of ~2.15 Å during the 30 ns simulation period. Molecular interactions analysis suggested that each inhibitor occupied the ATP-binding site of Cdk5/p25 and formed stable interactions. Finally, the binding free energy estimation suggested that each inhibitor had lowest binding energy than the reference compound (−113.10 kJ/mol) to recapitulate their strong binding with Cdk5/p25. Overall, these inhibitors could mitigate tau-mediated Alzheimer’s phenotype.

## 1. Introduction

Cyclin-dependent kinases (Cdks) play essential roles in post-translational modification of several proteins, thereby modulating their physiological fate. Cdks belong to the CMGC (Cyclin-dependent kinases, Mitogen-activated protein kinases, Glycogen synthase kinases and Cdk-like kinases) sub-family protein kinases and expedite the transfer of γ-phosphate from adenosine triphosphate (ATP) to peptide substrate(s) [1]. Cyclin-dependent kinase 5 (Cdk5, EC 2.7.11.22) is an atypical Cdk and is characterized as proline-directed serine/threonine protein kinase [2]. Formerly, Cdk5 was identified by biochemical purification from the bovine brain [3]. Unlike other Cdks that are physiologically activated by cyclins and regulate cell cycle progression, Cdk5 remains inactive in cell cycle. Instead, it is predominantly active in post-mitotic neuron(s) due to the restricted expression of its activators including p35 and p39 and/or their respective truncated forms, p25 and p29 [4]. Nevertheless, state-of-the-art cyclins such as cyclin-A, -D, and -E do not activate Cdk5, whereas in post-mitotic cells, Cdk5 is activated by cyclin-I [5,6].

Cdk5 regulates various neuronal functions including physiological development of the central nervous system (CNS), neurogenesis, neuronal migration, synaptic remodeling, synaptic activity, and learning and memory [7,8,9]. Mutation analyses suggested that Cdk5^−/−^ mice die just before or after birth due to abnormal corticogenesis, neuronal pathology, and detained cerebral cortex development [10]. Cdk5 also regulates several signaling pathways involved in plasticity and learning. For instance, postsynaptic density 95 (PSD-95) is a protein that structurally bridges signaling complexes and receptors at synapses. Cdk5-depenedent phosphorylation of PSD-95 prevents mulitmerization and clustering with ion channels, which subsequently maintains the morphological integrity of postsynaptic terminal [11]. Additionally, Cdk5 regulates synaptic plasticity and control neuronal and behavioral stimulus-induced excitability in neurons [12].

To date, numerous studies have supported the role of Cdk5 in several pathological phenotypes including cancer, neurodegenerative diseases, and ischemia. Abnormal regulation of Cdk5 by truncated activators (p25 and p29) contribute to neurodegeneration and has been implicated in Alzheimer’s diseases (AD), amyotrophic lateral sclerosis (ALS), Parkinson’s disease, Niemann–Pick type C disease, and Ischemia [13,14]. Cleavage of p35 to p25 by calpain forms Cdk5/p25 and hence potentiates tau protein hyperphosphorylation [15]. Tau proteins are microtubule-associated proteins, thereby stabilizing microtubule dynamics [16]. Hyperphosphorylation of tau proteins facilitate their self-assembly to form neurofibrillary tangles in AD brain, which eventually leads to neuronal dysfunction and cell death [17,18]. Consistent with these studies, several reports investigated that p25-overexpressing transgenic mouse models exhibit tau hyperphosphorylation and aggregation [19]. Recently, it has been shown that p25 overexpressed in the brain of JNPL3 mice harboring a human transgene at position P301L. Thereafter, the inhibition of calpain reduced p25 levels and attenuated tauopathy in these mice [20]. Cdk5 has been found to be deregulated in ALS patients, where it induces the hyperphosphorylation of neurofilament proteins and ultimately leads to apoptosis and neuronal cell death [21]. Recently, it has revealed that abnormal Cdk5 activity is associated with the pathogenesis of mutant superoxide-dismutase 1 (SOD1)-mediated ALS where it results in progressive death of motor neuron and paralysis [22]. Cdk5 implication in ALS suggested that Cdk5/p25 complex hyperphosphorylates neurofilament protein NF-H, a hallmark feature of ALS [23].

To this end, the pharmacological inhibition and/or targeted knockdown of Cdk5 relieved neurotoxicity and tau pathology [24]. Likewise, the disruption of NR2B–Cdk5 interactions can enhance memory formation and improved synaptic transmission [25]. Cdk5 inhibition has high therapeutic potential to prevent neuronal injury during stroke, brain injury, and high-risk surgeries [26,27]. Inhibition of Cdk5 after hypoxia/ischemic insult in injured rat models reduces infarct size and enhances functional recovery in neonatal rats [28]. Administration of indolinone-A inhibits Cdk5, thereby, activates stress responsive proteins which may protect neurons against further neuronal damage [29]. A study suggested that Cdk5 inhibitor (R)-roscovitine showed neuroprotective effects in transient model of focal cerebral ischemia in rats [30].

Nonetheless, available Cdk5 inhibitors may not be clinically translatable drugs to potentiate treatment platform in human neurological disorders [31]. Consequently, a robust need exists to design and develop Cdk5 inhibitors that are safe, efficient, and possess favorable drug-like properties. To this end, we have employed computational approaches and identified pyrrolidine-2,3-dione derivatives as novel inhibitors of Cdk5/p25. Herein, a pharmacophore model was generated from the previously known inhibitors of Cdk5 and eventually employed as 3D query in virtual screening of drug-like databases to identify competent drug-like hit molecules against the Cdk5/p25. Thereafter, the application of docking simulation, molecular dynamics simulation, and binding free energy calculations identified four pyrrolidine-2,3-dione derivatives as novel candidate inhibitors of Cdk5/p25. Unlike the training set compounds where the purine is the essential scaffold moiety of all the inhibitors, our results identified pyrrolidine-2,3-dione as novel scaffold moiety that could inhibit Cdk5/p25.

## 2. Material and Methods

### 2.1. Data Set Preparation and Pharmacophore Model Generation

The inhibitors of Cdk5, investigated by the same biological assays, were identified from literature survey [32,33,34]. The selected inhibitors of Cdk5 were assigned as training set and their 2D structures were drawn in Accelrys Draw v4.2 (Accelrys Inc., San Diego, CA, USA). Subsequently, the 2D structure of each compound was converted to their correspondent 3D structure in Discovery Studio (DS) v4.5 (BIOVIA, San Diego, CA, USA) [35]. The training set compounds were energy minimized by *Minimize Ligands* module, implanted in DS. Thereafter, *Common Feature Pharmacophore Generation* module of DS was employed to generate pharmacophore hypotheses. *Common Feature Pharmacophore Generation* module uses the *HipHop algorithm* and determines the atom-based critical common chemical features of all the compounds of a given training set to create 3D-pharmacophore models [36]. The protocol was optimized as: the inter-feature distance was set to 2.00 Å, conformation generation parameters were set to *Best* and *Flexible* mode, and minimum and maximum number of features were set to 3 and 6, respectively [37]. Finally, the 3D-pharmacophore models were generated and classified on the basis of pharmacophore fit value.

### 2.2. Validation of Pharmacophore Model

Pharmacophore validation evaluates the potentiality of pharmacophore to identify target compounds [38]. Pharmacophore validation was carried out by Guner–Henry approach, which is in robust practice in computational drug designing [39]. Herein, a dataset was formulated by collecting: (a) the already tested inhibitors of Cdk5 as active compounds, and (b) compounds that could not inhibit Cdk5 as the inactive molecules and designed a decoy test set. The selected pharmacophore was used as a 3D query to screen the decoy test set by *Ligand Pharmacophore Mapping* protocol, implanted in DS. The pharmacophore-mapped molecules were used as input values and parameters like goodness score (GH), enrichment factor (EF), percent ratio of actives (%A), percent yield of actives (%Y), false negative, and false positive were measured by equations:

(1) $$GH=\left\{\frac{\left[H_{a}(3A+H_{t})\right]}{4H_{t}A}\times \left[1-\frac{\left(H_{t}-H_{a}\right)}{\left(D-A\right)}\right]\right\}$$

(2) $$EF=\left(\frac{\frac{H_{a}}{H_{t}}}{\frac{A}{D}}\right)$$

where GH: Guner–Henry score (goodness score); EF: Enrichment Factor score; H_a_: number of actives in hit list; H_t_: total number of hits retrieved by pharmacophore; D: total number of compounds in decoy test set; A: total number of actives in decoy test set.

### 2.3. Drug-Like Database Designing and Virtual Screening

Since, drug-like properties are pre-requisites for a chemical compound to be used as a drug; therefore, the selected chemical databases (NCI, Asinex, and Specs databases) were filtered by *Lipinski’s Rule of Five* and *ADMET Descriptors* modules of DS to identify the drug-like compounds. Lipinski’s rule of five evaluates the physiochemical properties of chemical compounds [40]. The ADMET descriptors estimate the pharmacokinetics and pharmacodynamics properties such as absorption, distribution, metabolism, excretion, and toxicity (ADMET) of the drug-like molecules.

The validated pharmacophore was employed as a 3D query and each drug-like database was screened by *Ligand Pharmacophore Mapping* protocol, implanted in DS. The screening was carried out under the *Best/Flexible* parameterization environment at the *Maximum Omitted Features* “0”, which ensures the mapping of hit molecules onto all the features of the pharmacophore [41].

### 2.4. Molecular Docking Simulation

Molecular docking is a state-of-the-art approach in computational biology to identify and evaluate molecular interactions between the ligands and receptors [42,43,44,45]. Since, *Genetic Optimization of Ligand Docking* (GOLD v5.2.2; The Cambridge Crystallographic Data Centre, Cambridge, UK) package allows full flexibility of ligands and partial flexibility of protein; therefore, it estimates more reliable calculations in docking simulations [46]. Herein, GOLD package was used to dock the pharmacophore-retrieved drug-like molecules into the active site of Cdk5/p25. The structure of human Cdk5 in complex with p25 (Cdk5/p25) and Roscovitine was taken from protein data bank (PDB) and was prepared for docking by removing unwanted molecules. The inbuilt module of GOLD package (*add hydrogen*) was used to add hydrogen atoms to Cdk5/p25. The ligand binding site of Cdk5 was defined within a radius of 7.00 Å of the inbound inhibitor (Roscovitine). The Goldscore and ASP (Astex Statistical Potential) scoring functions were used as the default scoring and rescoring functions, respectively [47]. The Goldscore is the original fitness function, optimized for ligand position prediction, and is the default scoring function in GOLD. It relies on factors such as hydrogen bond energy, van der Waals energy, and ligand torsion strain. The ASP rescoring function of GOLD measures the atom–atom potential and has comparable accuracy to the Chemscore and Goldscore fitness functions [48]. The Cdk5-bound inhibitor (Rescovitine) was used as the reference compound throughout the analyses. The drug-like molecules with highest Goldscore and ASP scores, highly stable conformation (clustering analysis), and the formation of hydrogen bonds with the catalytic active residue(s) of Cdk5 were selected. Since, chemical synthesis of compound(s) is an expensive process; hence, only those drug-like molecules were shortlisted that are commercially available.

### 2.5. Molecular Dynamics (MD) Simulation

From docking analysis, the true positive candidate inhibitors (successfully docked candidate hits) of Cdk5/p25 were subjected to molecular dynamics simulation to explore their stability and mechanism of interaction under the simulated physiological environment. For each drug-like molecule, independent simulation system was prepared in *Groningen Machine for Chemical Simulation* (GROMACS v5.1.4) package [49]. The parameters for protein coordinates and topology were created by CHARMm36 all atoms force field [50], whereas, for the ligands, the topology was generated by SwissParam [51]. Each system was simulated in octahedral box and solvated with TIP3P water model [52]. Periodic boundary conditions were applied in all directions to mimic the infinite system. In order to simulate physiological pH, each system was buffered with 0.1 M NaCl solution. Thereafter, each system was subjected to an initial preparatory phase including energy minimization and equilibration. Energy minimization was conducted at a maximum force of 10 kJ/mol to avoid steric clash and bad contacts. During equilibration, the configurational status of candidate inhibitors and Cdk5/p25 backbone atoms were preserved by all atoms position restraints. Equilibration was performed in two stages: first, temperature equilibration was carried out under an NVT ensemble (at constant number of particles, volume, and temperature) for 100 ps at 300 K while using V-rescale thermostat. Second, NPT ensemble was equilibrated at constant number of particles, pressure, and temperature at 1.0 bar by Parrinello–Rahman barostat. Afterwards, each equilibrated system was escalated to a freely movable molecular dynamics simulation. The particle mesh ewald (PME) method was adopted to measure the long-range electrostatic interactions using a 10 Å cut-off distance. The bond distances were restrained using *LINCS algorithm* which allowed 2 fs time step in all simulations. During simulation, V-rescale thermostat and Parrinello–Rahman barostat were employed to sustain the temperature and pressure at 300 K and 1.0 bar, respectively.

### 2.6. Binding Free Energy Calculations

The binding free energies (ΔG_bind_) of the reference compound and final candidate hits with Cdk5/p25 were calculated by molecular mechanics Poisson-Boltzmann surface area (MM-PBSA) method [53]. The binding free energy of a protein-ligand complex (ΔG_bind_) in solution is defined as:

(3) $$\Delta G_{bind}=G_{complex}-\left[G_{protein}+\;G_{ligand}\right]$$

A molecular dynamics simulation generates an ensemble of time-equidistance conformations. The free energy term is calculated as an average over the representative structures as:

(4) $$\left(G)=E_{MM}+\Delta G_{sol}-T(S_{MM}\right)$$

The energetic term E_MM_ is defined as:

(5) $$E_{MM}=E_{int}+E_{coul}+E_{LJ}$$

where *E_int_* indicates bond, angle, and torsional angle energies, and *E_coul_* and *E_LJ_* display the intermolecular electrostatic and van der Waals energies, respectively.

The solvation term G_solv_ is the combination of G_polar_ and nonpolar contribution, G_nonpolar_:

(6) $$\Delta G_{solv}=\Delta G_{polar}+\Delta G_{nonpolar}$$

The non-polar contribution G_nonpolar_ is proportional to the solvent accessible surface area (SASA):

(7) $$G_{nonpolar}=\gamma \left(SASA\right)+\beta$$

where γ = 0.0227 kJ mol^−1^Å^−2^ and β = 3.849 kJ mol^−1^.

Herein, an ensemble of thirty time-equidistant snapshots of candidate inhibitors in complex with Cdk5/p25 were taken from the entire MD trajectories and the binding free energy calculations were performed.

## 3. Results and Discussion

### 3.1. Ligand-Based Pharmacophore Generation

**Figure 1:**
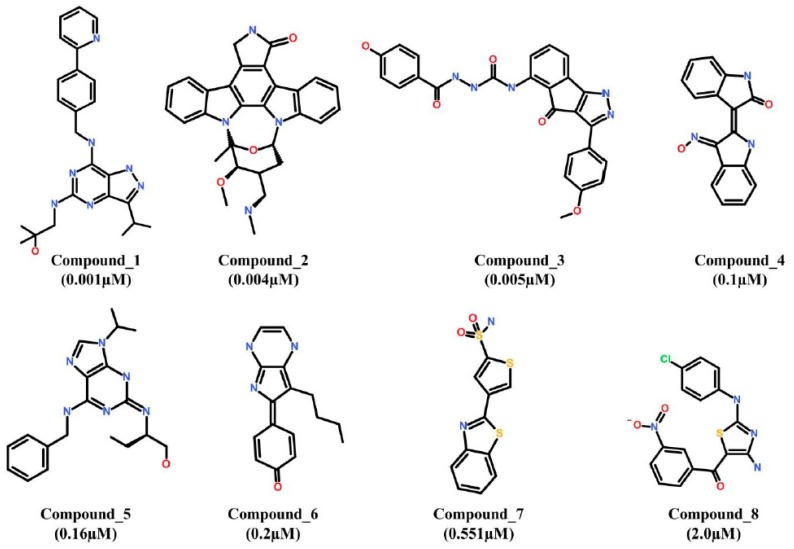
Training set compounds. The 2D chemical structures and IC_50_ (µM) values of the training set compounds for hypotheses generation.

Ligand-based pharmacophore modeling is a well-established approach in computational drug discovery processes. Herein, eight chemically diverse inhibitors of Cdk5 were collected from literature mining [32,33,34]. The selected inhibitors were preferred for their symmetry in inhibitory mechanism of Cdk5 and same biological assays. The inhibitory activity range of the selected inhibitors was 0.001–2.0 µM. The 2D structures of all the selected inhibitors were drawn and were assigned as training set (Figure 1).

Thereafter, the training set compounds were energy minimized by CHARM force field to obtain the lowest energy conformation [54,55]. The global chemical features of the training set compounds were determined by *Feature Mapping* module, implanted in DS. It was observed that the training set compounds possess hydrogen bond acceptor (HBA), hydrogen bond donor (HBD), hydrophobic (HYP), hydrophobic aromatic (HYA) and ring aromatic (RAR). Afterwards, the *Common Feature Pharmacophore Generation* module of DS was employed and ten pharmacophore models were generated. Our results showed that the ranking scores of hypotheses ranged from 55.82 to 75.17 kcal/mol (Table 1).

**Figure 2:**
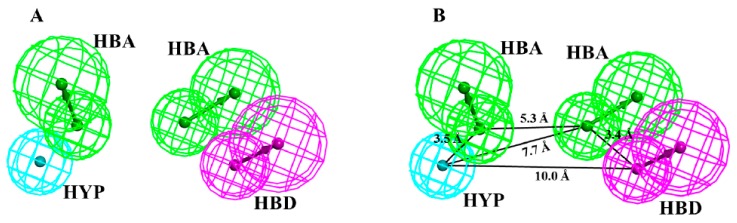
Description of pharmacophore model (Hypo1). The chemical space of the selected pharmacophore is comprised of two hydrogen bond acceptors—**HBA** (green), one hydrogen bond donor—**HBD** (magenta), and one hydrophobic—**HYP** (cyan) feature. The inter-feature distance constraints are shown in angstrom (Å).

The first eight hypotheses were four featured, while the last two were three featured hypotheses. Furthermore, the first four hypotheses were comprised of two hydrogen bond acceptor (HBA) features, one hydrogen bond donor (HBD) feature, and one hydrophobic (HYP) feature (Table 1). Such combination of pharmacophoric features indicated that the selected compounds had high resemblance of interaction mechanism with Cdk5. Our results suggested that the highly ranked hypothesis was scored 75.17 kcal/mol. Since, the first two hypotheses had the same interaction features and nearly equal ranking scores (Table 1). Therefore, we superimposed the two hypotheses and examined their spatial differences. It was observed that both the hypotheses were entirely overlaid each other by a maximum root mean square deviation (RMSD) value of 0.51 Å and no significant difference was observed (Figure S1). Subsequently, Hypo1 was chosen as a 3D query for further analyses. Our results revealed that Hypo1 is comprised of a total of four chemical features (Figure 2).

The detailed study of Hypo1 suggested that polar interactions are the pre-dominant features and delineates the chemical space of the selected pharmacophore. The four features of Hypo1 was composed of two hydrogen bond acceptors (HBA), one hydrogen bond donor (HBD), and one hydrophobic (HYP) feature, respectively (Figure 2A). The spatial orientation of the pharmacophoric features of Hypo1 showed that two polar interaction features (HBA and HBD) were coupled and closely oriented at an inter-feature distance of 3.4 Å (Figure 2B). The other polar interaction was distant lodged by an inter-feature distance of 5.3 Å from the co-partner HBA feature of the coupled polar interactions (Figure 2B). Furthermore, the non-polar interaction was also distant settled in the closed vicinity of the second polar interaction (HBD) by an inter-feature distance of 10.0 Å.

### 3.2. Quality Assessment Test (Validation) of Pharmacophore (Hypo1)

The potentiality of Hypo1 was evaluated to differentiate between the active and inactive inhibitors of Cdk5/p25. A decoy test set was prepared, which was comprised of 40 active inhibitors of Cdk5 (A) and 668 inactive molecules of Cdk5 (Table 2).

During the screening of decoy test set by Hypo1, 46 compounds (H_t_) were mapped, where 38 compounds were the active inhibitors (H_a_) of Cdk5. Our results suggested that Hypo1 mapped 95% active inhibitors and obtained the highest percent yield of 82.6% (Table 2). Furthermore, Hypo1 showed high GH score of 0.84 and enrichment factor (EF) value of 14.62 (Table 2). Decoy test validation is an established approach in pharmacophore validation and the Hypo1 showed parallel results with the published report [56]. The goodness of fit (GH) score ranges between 0 and 1, which indicates a null model and an ideal model, respectively [57]. Since, several studies have already been reported that a pharmacophore with higher GH score is more reliable for virtual screening [39,56]. Therefore, we suggest that the resultant pharmacophore may screen the candidate hits that harbor the key complementary pharmacophoric features of the ATP-binding site of Cdk5/p25.

### 3.3. Development of Drug-like Database and Virtual Screening

**Figure 3:**
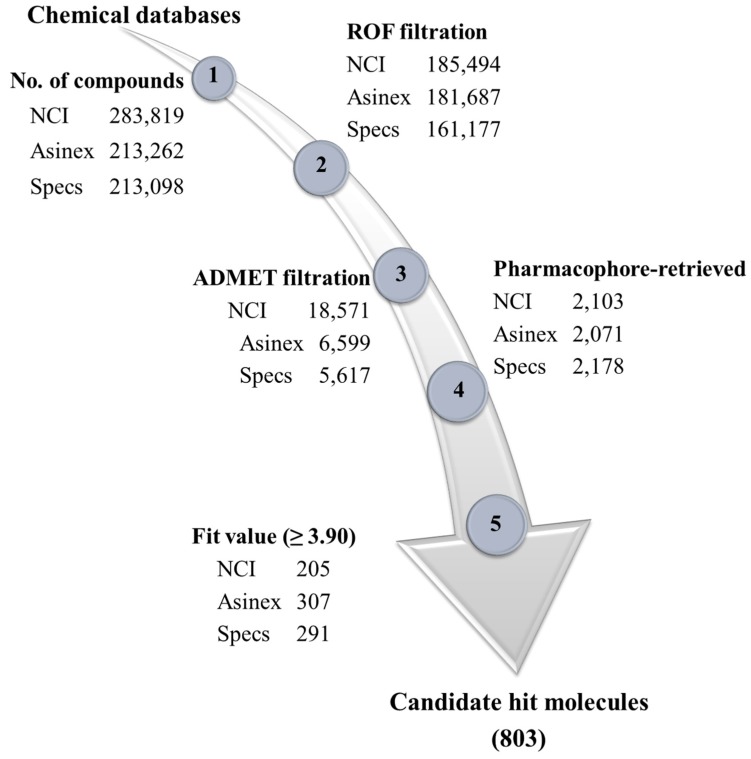
Drug-like database development and virtual screening. Chemical database are included NCI, Asinex, and Specs databases. Lipinski’s rule of five and ADMET descriptors tests filtered drug-like molecules. Pharmacophores-based virtual screening identified hit compounds against Cdk5/p25.

A chemical compound is used as a drug-like molecule if its physiochemical, pharmacodynamics, and pharmacokinetics properties exhibit drug-like properties [41]. Herein, *Lipinski’s Rule of Five* (ROF) was used to profile the physiochemical properties of three chemical databases including NCI, Asinex, and Specs databases. According to ROF, a drug-like compound should be membrane permeable and could easily be absorbed if it has total number of hydrogen bond acceptors and hydrogen bond donors less than 10 and 5, and molecular weight and AlogP values are less than 500 Da and 5, respectively [40]. Our results suggested that ROF filtered 185494, 181687, and 161177 compounds from NCI, Asinex, and Specs databases, respectively (Figure 3).

Likewise, the pharmacodynamics and pharmacokinetics properties of the drug-like molecules are critical parameters of drug discovery in terms of absorption, distribution, metabolism, excretion, and toxicity. Accordingly, the drug-like compounds filtered by ROF were subsequently subjected to *ADMET Descriptors* module in DS. Herein, the drug-like compounds were evaluated for their potentiality to cross blood-brain barrier, solubility, hepato-toxicity, cytochrome toxicity, and absorption (Figure 3). Finally, a total of 18,571, 6599 and 5617 compounds were retrieved from NCI, Asinex, and Specs databases as drug-like molecules, respectively (Figure 3). Pharmacophore-based virtual screening is an established approach in drug discovery [39,41]. Combined pharmacophore-based screening and fit value filtration retrieved 803 drug-like molecules as the candidate hits of Cdk5/p25 (Figure 3).

### 3.4. Molecular Docking Simulation

Molecular docking is a reliable and efficient technique to evaluate protein–ligand interaction and remained the essential procedure of several drug designing strategies [43,44,45,58]. In order to identify the true positive candidate hits of Cdk5/p25, molecular docking of the candidate hits was carried out in the active site (ATP-binding site) of Cdk5/p25. The structure of Cdk5/p25 in complex with Roscovitine and resolution of 2.2 Å was taken from Protein Data Bank (PDB ID: 1UNL) (Figure S2) [32]. The docking of Roscovitine (hereafter REF) obtained highest Goldscore and ASP scores of 67.67 and 26.32, respectively (Table 3). Consequently, the docking scores of the REF was used as cut-off value and the best candidate inhibitors of Cdk5/p25 were identified. The candidate inhibitors were further inspected for their conformational stability and hydrogen bond interactions with the catalytic active residues of Cdk5/p25. We argued that candidate inhibitors that formed stable clusters and hydrogen bonds with either of Ile10, Phe80, Glu81, Phe82, Cys83, Gln85, Asp86, and Asn144 residues of Cdk5, might be the proposed candidate inhibitors of Cdk5/p25 [32,59]. Finally, the inspection of candidate inhibitors for their commercial availability, a total of 12, 38, and 41 compounds were isolated from NCI, Asinex, and Specs databases, respectively (Table S1). The Goldscore and ASP score values of the final hits are given in Table 3.

### 3.5. Molecular Dynamics Simulation

The candidate inhibitors successfully docked in the ATP-binding site of Cdk5/p25 were subjected to 30 ns MD simulation. Herein, several parameters were used to evaluate the molecular interactions, conformational stability, and the binding orientation of the final inhibitors of Cdk5/p25. Our observation suggested that the REF compound as well as all the candidate inhibitors occupied the ATP-binding site of Cdk5/p25 (Figure S3). The ATP-binding site of Cdk5/p25 is sub-divided into Gly-rich loop, activation loop, and the hinge region. Previously, it has been investigated that small molecule inhibitors occupied and oriented between the sub-pockets of the ATP-binding site of Cdk5/p25 [32,33,59].

#### 3.5.1. Root Mean Square Deviation (RMSD) Analysis

**Figure 4:**
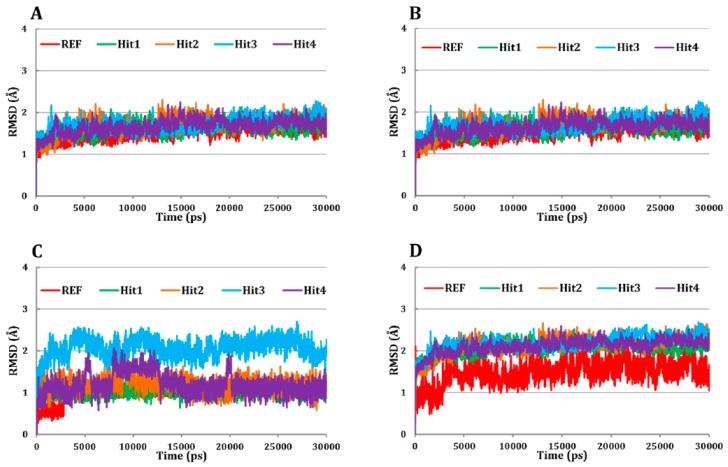
Root mean square deviation (RMSD) analyses.(**A**) RMSD of the C_α_ atoms of Cdk5/p25 in all the systems revealed their binding stability. (**B**) RMSD of backbone atoms of the Cdk5/p25 suggested their conformational stability in simulated environment. (**C**) RMSD of each simulated inhibitor (REF and hit molecules) suggested their stability during the simulation. (**D**) RMSD of the Cdk5/p25 in complex with ligand(s) advocated ligand(s) binding and stability during the entire simulation period. Red, green, orange, cyan, and blue colors represent REF, Hit1, Hit2, Hit3, and Hit4, respectively.

The RMSD analysis of C_α_ (carbon alpha) atoms of Cdk5/p25 suggested that all the systems were converged after 4 ns simulation (Figure 4A). The RMSD value of C_α_ atoms of Cdk5/p25 in all the systems ranged from ~1.3–1.9 Å suggested that each system remained stable during the entire production phase (Figure 4A).

Furthermore, the backbone RMSD of each system suggested that each system behaved normal during the entire simulation period (Figure 4B). Similarly, the RMSD analysis of the REF compound, and all the hit molecules suggested that all the tested inhibitors obtained lowest RMSD values (<2.5 Å) and behaved stable during the production phase of simulation (Figure 4C). Next, we addressed whether the REF compound and/or the hit molecules remained stable in the ATP-binding site of Cdk5/p25 during the production phase? Our analysis suggested that the RMSD of each candidate inhibitor in Cdk5/p25-hit(s) complex remained stable during the production phase (Figure 4D). Average values of the RMSD of C_α_ atoms, backbone atoms, tested inhibitors, and Cdk5/p25-inhibitor(s) complex are given in Table 3.

#### 3.5.2. Molecular Overlay and Molecular Interaction Analysis

**Figure 5:**
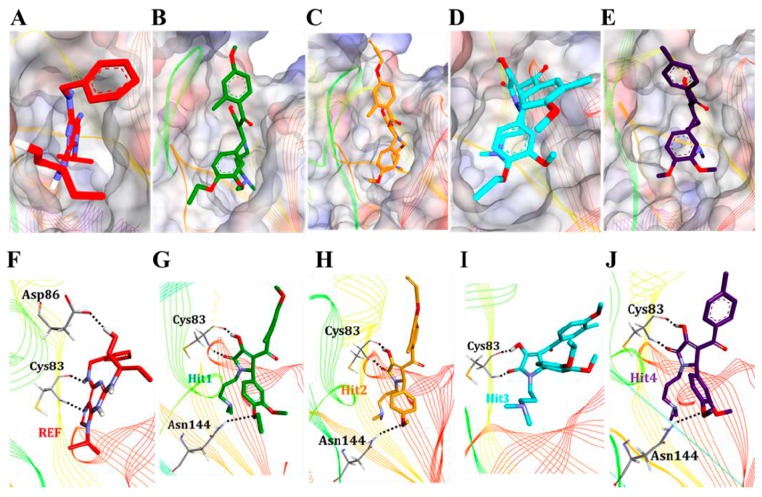
Binding mode analyses. All the hit molecules preferentially lodged in the ATP-binding site of Cdk5 with almost similar molecular orientation (**A**–**E**). The REF, Hit1, Hit2, Hit3, and Hit4 are depicted as red, green, orange, cyan, and blue, respectively. The three dimensional (3D) molecular interaction pattern of the REF and all the hit molecules with Cdk5/p25 has been illustrated (**F**–**J**). Interacting residues are displayed as thin sticks and labeled. REF, Hit1, Hit2, Hit3, and Hit4 are depicted as thick stick representation and colored as red, green, orange, cyan, and blue, respectively. Hydrogen bonds have been shown as black-dashed lines.

**Figure 6:**
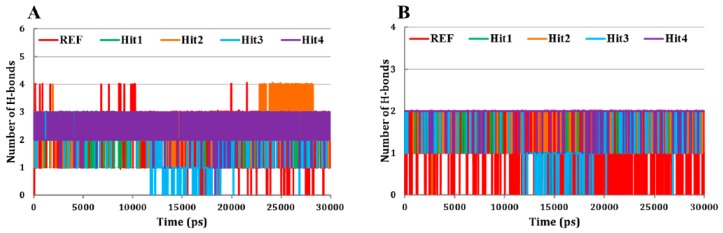
Evaluation of hydrogen bond formation between the Cdk5/25 and hit molecules. (**A**) The REF compound as well as all the hit molecules formed hydrogen bonds with Cdk5/p25. All the hit molecules showed slightly higher number of hydrogen bonds. (**B**) Hydrogen bonds remained persistent between the Cys83 of Cdk5 and each hit molecule. Red, green, orange, cyan, and blue colors represent REF, Hit1, Hit2, Hit3, and Hit4, respectively.

Since, it has already been reported that small molecule inhibitors occupied the ATP-binding site of Cdk5/p25 [32,59]. Therefore, we examined the molecular overlay of the REF compound and hit molecules in Cdk5/p25. Our results showed that all the tested inhibitors occupied the ATP-binding site of Cdk5/p25 (Figure 5A–E). Our results emphasized that the REF compound as well as all the hit molecules obtained almost similar conformational orientation in the ATP-binding site of Cdk5/p25. Furthermore, polar and non-polar interactions play a critical role in protein-drug interaction [32,59,60,61]. Therefore, we argued to identify the nature of molecular interactions between the Cdk5/p25 and each hit molecule. The hydrogen bond (H-bond) analysis suggested that all the hit molecules formed hydrogen bonds with Cdk5/p25 (Figure 5F–J and Figure 6A). Our results strongly followed the previous reports that hydrogen bonds are established between the Cdk5/p25 and the corresponding inhibitors [32,59,62].

Furthermore, we focused on polar interactions (H-bond formation) of hit(s) with the Cys83 residue of Cdk5, which has already been investigated as the potential mechanism of Cdk5/p25 inhibition [32,59,62]. Interestingly, our results suggested that the REF compound formed H-bond with the Cys83 residue of Cdk5 and was also considered as a validation of our methodology (Figure 5F and Figure 6B). Consequently, we emphasized that the candidate hits should form H-bonds with the Cys83 residue of activated Cdk5 (Cdk5/p25). Luckily, the final hit compounds also formed H-bonds with the Cys83 of Cdk5 (Figure 5G–J, Table S2).

Likewise, we addressed whether H-bonds between the Cys83 of Cdk5 and the REF compound and/or hit molecules are consistent throughout the production phase. Fortunately, our results demonstrated that all the hit molecules showed stable consistency of the H-bond formation with the Cys83 residue of Cdk5 throughout the production phase (Figure 6B). Since, other Cdks have also been investigated for their mode of inhibition [34,63]. Therefore, we asked the importance of hydrogen bond formation at position Cys83 in Cdk5. In parallel with Cdk5, Cdk9 has cysteine (Cys106) at the same topological position. Interestingly, the Cys106 residue of Cdk9 forms hydrogen bond interaction with the inhibitor (S)-CR8 to trigger Mcl-I down regulation in neuroblastoma cells [64]. In another study, members of 4-(thiazol-5-yl)-2-(phenylamino)-pyrimidine-5-cabonitrile series also suggested hydrogen bond formation with the Cys106 residue of Cdk9 [65]. Our results also followed the findings of several other reports of the formation of H-bond between the residue at this particular position (Cys83 in Cdk5) and their correspondent inhibitor(s). For instance, leucine at position 83 (Leu83) of Cdk2 occupied the same topological position and has established hydrogen bond interaction(s) with the bound inhibitors [34,63]. Cdk1 also incorporated leucine at position 83 (Lys83) and forms H-bonds with its ATP-competitive inhibitor [66]. Furthermore, Cdk8 has coded alanine at this topological position and forms H-bond with its selective inhibitor [67]. Our analyses also observed that each hit molecule formed an additional H-bond with Asn144 residue of Cdk5 (Figure 5G–J, Table S2). In parallel, the formation of hydrogen bonds by inhibitor(s) with the ATP-binding site residues of Cdk5 other than Cys83 has frequently been observed [33,59,62].

Since, non-polar interactions play a significant role in protein–drug interaction [33,59,60,68]. Therefore, we argued to explore non-polar interactions between the Cdk5/p25 and each hit molecule. Our analyses investigated that all the hit molecules formed non-polar interactions with the ATP-binding site residues of Cdk5/p25 (Table S2). Our findings of non-polar interactions established by hit molecules with the ATP-binding site residues of Cdk5/p25 are in parallel with published literature [32,33,59,62]. Overall, these results suggested that the newly identified hit molecules could potentially inhibit Cdk5/p25.

### 3.6. Binding Free Energy Analysis

**Figure 7:**
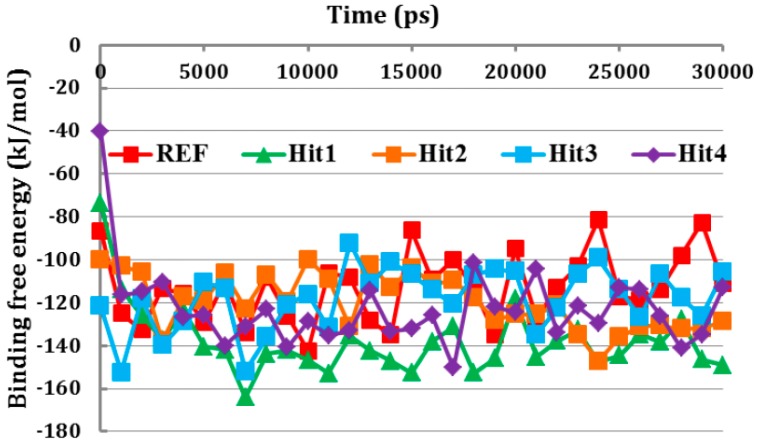
Binding free energy calculation. The GROMACS tool “g_mmpbsa” has calculated the binding free energy between the Cdk5/p25 and each hit molecule. All the hit molecules obtained lowest binding free energy values than the REF compound. Red, green, orange, cyan, and blue colors depict REF, Hit1, Hit2, Hit3, and Hit4, respectively.

Since, the REF compound and final hit molecules showed stable RMSD values and strong molecular interactions with Cdk5/p25. Therefore, we argued to identify the candidate hits with strong binding affinity towards Cdk5/p25. To this end, the binding free energy analysis of the REF compound and each hit molecule was carried out against the Cdk5/p25 complex. MM/PBSA is a well-established approach to explore binding free energy between the protein and ligand(s) [53]. Our results suggested that all the newly identified hit molecules obtained lowest binding free energy than the REF compound (Figure 7).

Based on binding free energy analysis, our results proclaimed that the hit molecules had higher affinity towards Cdk5/p25 and may strongly inhibit Cdk5/p25. The ΔG values for all the tested compounds were in the range of −113.10 to −137.90 kJ/mol. The average binding free energy value of each candidate hit is given in Table 3. The 2D structure and SMILE ID of candidate hits have been depicted in Figure S4.

## 4. Conclusions

The abnormal production of p25-mediated hyperactive cyclin-dependent kinase 5 (Cdk5) results in aberrant hyperphophorylation of tau protein, neuroinflammation, and Alzheimer’s phenotypes. Therefore, the inhibition of Cdk5/p25 complex may relieve the aberrant phosphorylation of tau protein to mitigate AD pathology. Herein, computational simulations have identified pyrrolidine-2,3-dione derivatives as the candidate inhibitors of Cdk5/p25 complex to alleviate AD pathologies. A ligand-based pharmacophore was generated and validated by decoy test set method. The validated pharmacophore retrieved drug-like compounds from chemical databases like NCI, Asinex, and Specs databases. Molecular docking approach filtered the drug-like molecules and identified the candidate hit compounds of Cdk5/p25. Molecular dynamics simulation and binding free energy calculation retrieved four hit molecules as the best candidate inhibitors of Cdk5/p25 complex. The root mean square deviation (RMSD) analysis of C_α_ atoms and backbone atoms of Cdk5/p25, and Cdk5/p25 in complex with hit molecules revealed that all the candidate molecules obtained lowest RMSD value (<2.5 Å) and behaved stable during the production phase of simulation. Furthermore, the final hit molecules occupied the ATP-binding site of Cdk5/p25 and formed consistent hydrogen bonds with the Cys83 residue the ATP-binding site of Cdk5/p25. Finally, MM/PBSA approach suggested that each candidate inhibitor had lowest binding free energy with the Cdk5/p25 complex to recapitulate their strong binding. Overall, we suggest that the inhibition of Cdk5/p25 by the newly identified pyrrolidine-2,3-dione derivatives may alleviate the tau-associated Alzheimer’s pathology.

## Figures and Tables

**Table 1:** Hypotheses generation. Pharmacophore characterization suggested that hydrogen bond acceptor (HBA), hydrogen bond donor (HBD), and hydrophobic (HYP) features are the pre-dominant features of Cdk5 inhibition.

Hypo. No.	Features ^a^	Rank ^b^	Direct Hit ^c^	Partial Hit ^d^	Max. Fit
1	HYP, HBD, HBA, HBA	75.175	11111111	00000000	4
2	HYP, HBD, HBA, HBA	74.277	11111111	00000000	4
3	HYP, HBD, HBA, HBA	70.057	11111111	00000000	4
4	HYP, HBD, HBA, HBA	68.758	11111111	00000000	4
5	HYP, HYP, HBD, HBA	64.341	11111111	00000000	4
6	HYP, HYP, HBD, HBA	63.638	11111111	00000000	4
7	HYP, HYP, HBD, HBA	62.807	11111111	00000000	4
8	HYP, HBD, HBA, HBA	61.279	11111111	00000000	4
9	HYP, HBA, HBD	57.531	11111111	00000000	4
10	HYP, HBD, HBA	55.820	11111111	00000000	4

^a^ Features: HYP—Hydrophobic; HBD—Hydrogen bond donor; HBA—Hydrogen bond acceptor; ^b^ Rank: The higher the ranking score the less likely the molecules fit the hypothesis by chance correlation. The best hypothesis shows the highest value; ^c^ Direct hit indicates whether (1) or not (0) a molecule in the training set mapped every feature in the hypothesis; ^d^ Partial hit indicates whether (1) or not (0) a particular molecule in the training set mapped all but one feature in the hypothesis.

**Table 2:** Pharmacophore validation. Decoy test method suggested that pharmacophore (Hypo1) is able to differentiate between the active and inactive molecules of Cdk5.

S. No.	Parameter	Calculated Value
1	Total number of molecules in the database (D)	708
2	Total number of active molecules of Cdk5 in the database (A)	40
3	Total number of active molecules of Cdk5 in the retrieved hits (H_a_)	38
4	Number of retrieved hits by pharmacophore (H_t_)	46
5	% Yield of actives ((H_a_/H_t_) × 100]	82.6
6	% Ratio of actives ((H_a_/A) × 100]	95.0
7	False positive (H_t_ − H_a_)	8
8	False negative (A − H_a_)	2
9	Goodness of fit (GH)	0.84
10	Enrichment factor (EF)	14.62

**Table 3:** Docking scores and the root mean square deviation (RMSD) values of the final hit molecules of Cdk5/p25.

Compound	Docking Score		RMSD (Å)				Binding Free Energy (kJ/mol)
	Goldscore ^#^	ASP ^$^	Complex	C_α_ Atoms ^ψ^	Backbone Atoms	Inhibitors	
**REF**	67.67	26.32	1.54	1.54	1.54	1.02	−113.10
Hit1	79.22	33.14	2.04	1.59	1.57	1.02	−137.90
Hit2	77.39	30.64	2.12	1.70	1.68	1.20	−119.65
Hit3	86.33	35.31	2.18	1.70	1.68	2.06	−118.22
Hit4	75.35	30.29	2.10	1.65	1.63	1.20	−122.65

**^#^** Goldscore—Goldfitness score; **^$^** ASP—Astex statistical potential; **^ψ^** C_α_ Atoms—Alpha carbon atoms of the Cdk5/p25.

## Supplementary Materials

The following are available online at https://www.mdpi.com/2077-0383/8/5/746/s1, Figure S1: Superimposition of the first two hypotheses, Figure S2: Docking site of Cdk5/p25 complex, Figure S3: Superimposition of the reference (REF) compound and candidate hits, Figure S4: 2D structure and SMILE IDs of the final candidate hits, Table S1: Docking results analysis, Table S2: Molecular interactions between the Cdk5/p25 and ligands, Table S3: IUPAC name, PubChem ID and Supplier information of the final hit compounds.
