# Supplementary material for: Computational Simulations Identify Pyrrolidine-2,3-Dione Derivatives as Novel Inhibitors of Cdk5/p25 Complex to Attenuate Alzheimer’s Pathology
Source: J Clin Med. 2019 May 24;8(5):746. doi: 10.3390/jcm8050746 (PMC6572193; doi:10.3390/jcm8050746)
Supplement: Supplementary file 1 [file jcm-08-00746-s001.pdf]

# Computational Simulations Identify Pyrrolidine-2,3-dione Derivatives as Novel Inhibitors of Cdk5/p25 Complex to Attenuate Alzheimer's Pathology

Amir Zeb[a], Donghwan Kim[a], Sayed Ibrar Alam[b], Minky Son[a], Raj Kumar[c], Shailima Rampogu[a], Saravanan Parameswaran[a], Rahul Mahadev Shelake[d], Rabia Mukhtar Rana[a], Shraddha Parate[a], Jae-Yean Kim*[d] and Keun Woo Lee*[a]

[a]*Division of Life Science, Division of Applied Life Science (BK21 Plus), Research Institute of Natural Science (RINS), Gyeongsang National University (GNU), 501 Jinju-daero, Jinju 52828, Republic of Korea*

[b]*Division of Life Sciences and Applied Life Science (BK 21plus), College of Natural Sciences, Gyeongsang National University (GNU), 501 Jinju-daero, Jinju 52828, Republic of Korea*

[c]*Institute of Chemical Processes (ICP), Seoul National University, 1 Gwanak-ro, Gwanak-gu, Seoul, 08826, Korea*

[d]*Division of Applied Life Sciences, Plant Molecular Biology and Biotechnology Research Center, Gyeongsang National University, Jinju 660-701, Korea.*

Running title: Pyrrolidine-2,3-dione Derivatives Inhibit Cdk5/p25 Complex

***Corresponding authors**

Keun Woo Lee (PhD)

Professor

Email: kwlee@gnu.ac.kr
kuku1004@gmail.com

Jae-Yean Kim (PhD)

Professor

Email: kimjy@gnu.ac.kr

# Supplementary Information

**Figure S1**

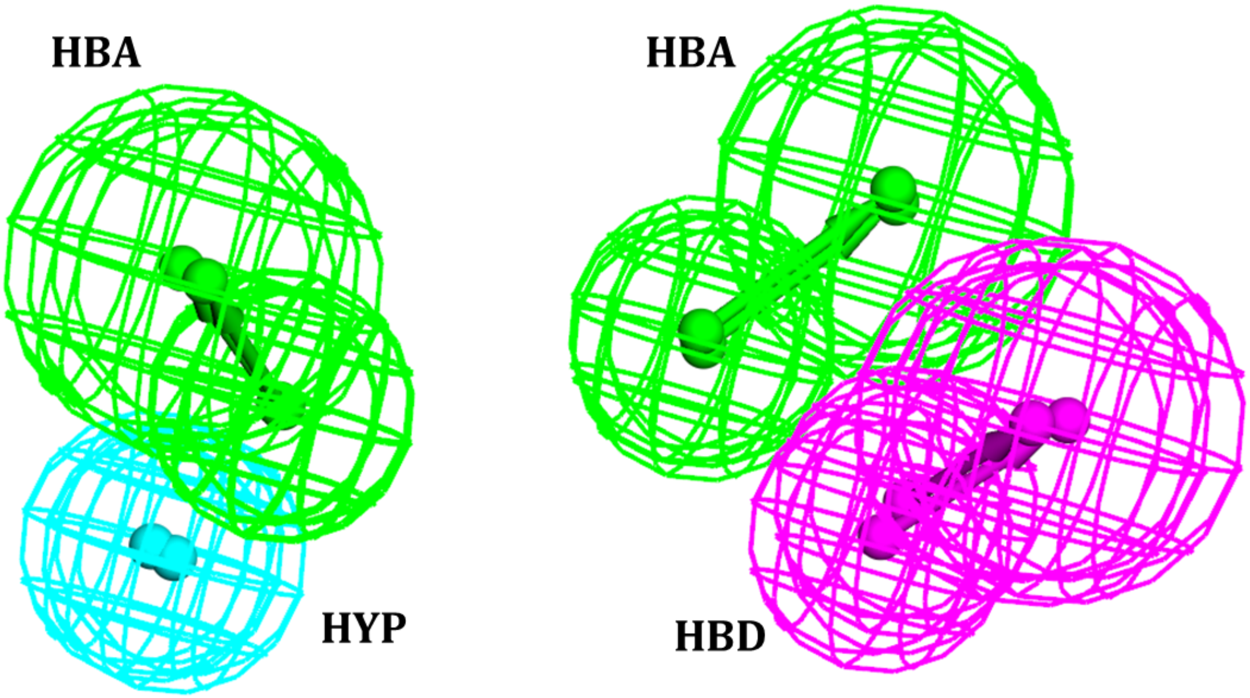

**Figure S2**

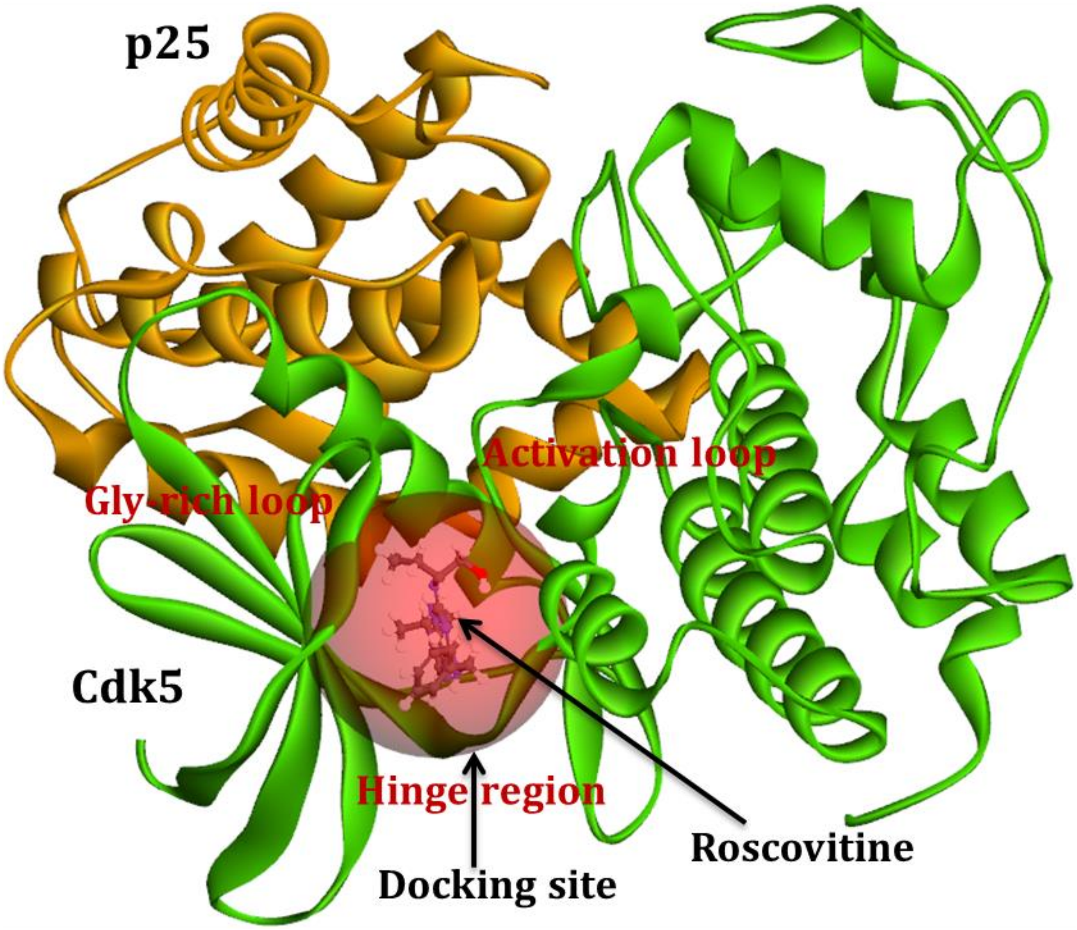
p25
Activation loop
Gly-rich loop
Cdk5
Hinge region
Roscovitine
Docking site

**Figure S3**

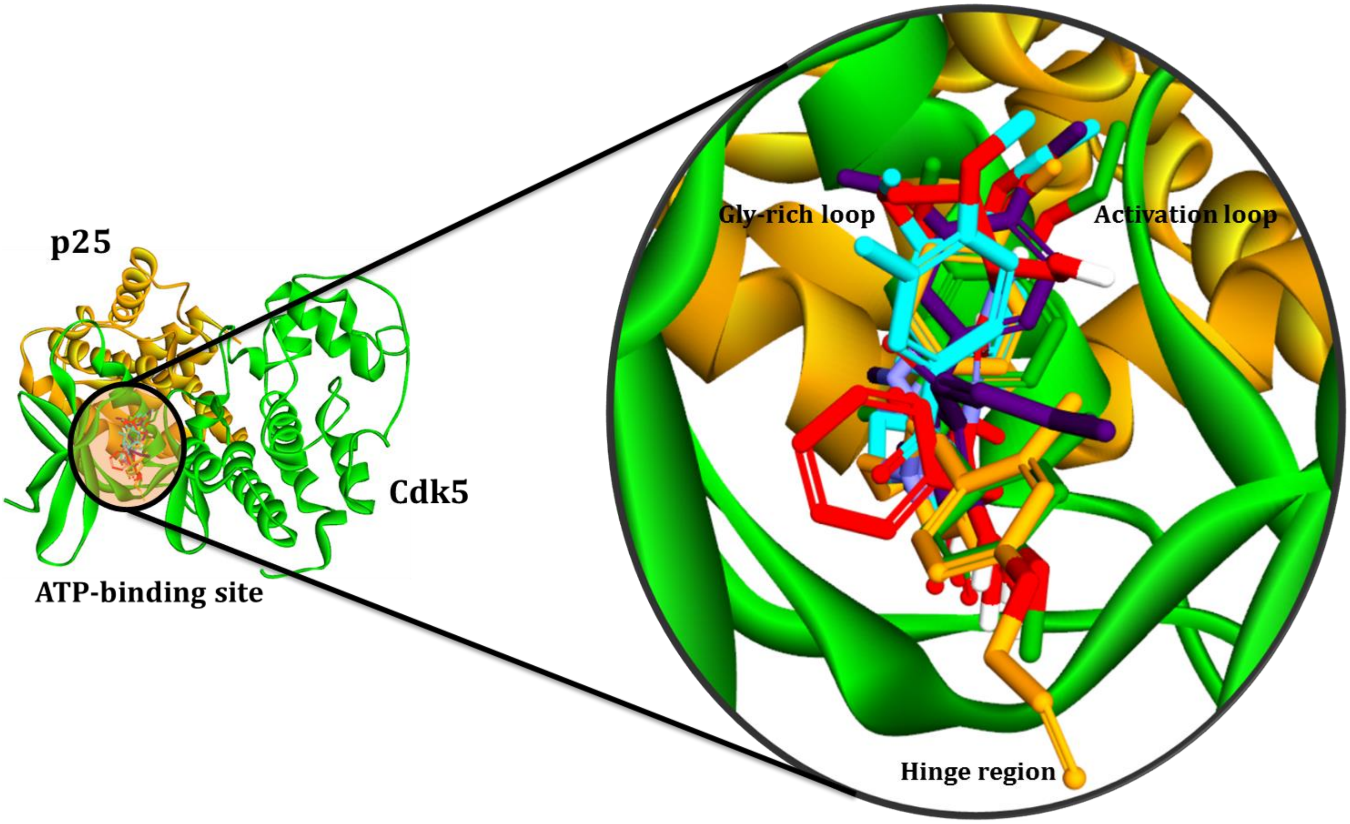


**Superimposition of REF, Hit1, Hit2, Hit3, and Hit4**

**Figure S4**

| 2D Structure | SMILE ID | 2D Structure | SMILE ID |
|---|---|---|---|
| 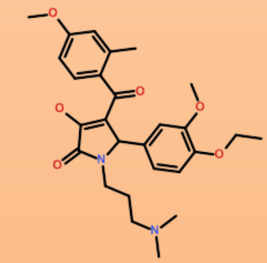 Hit1 | CCOc1ccc(cc1OC)C2N(CCCN(C)C)C(=O)C(=C2C(=O)c3ccc(OC)cc3C)O | 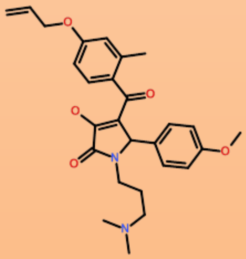 Hit2 | COc1ccc(cc1)C2N(CCCN(C)C)C(=O)C(=C2C(=O)c3ccc(OCC=C)cc3C)O |
| 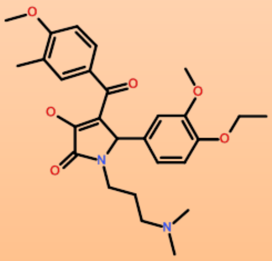 Hit3 | CCOc1ccc(cc1OC)C2N(CCCN(C)C)C(=O)C(=C2C(=O)c3ccc(OC)c(C)c3)O | 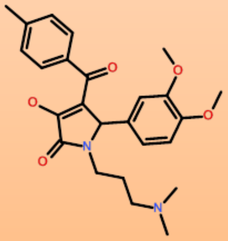 Hit4 | COc1ccc(cc1OC)C2N(CCCN(C)C)C(=O)C(=C2C(=O)c3ccc(C)cc3)O |

**Table S1.** Docking results analysis.

| Parameter | NCI | Asinex | Specs | Total |
|---|---|---|---|---|
| **Compounds** | 205 | 291 | 307 | 308 |
| **ChemPLP Score (≥ 67.67)** | 56 | 143 | 127 | 326 |
| **ASP Score (≥ 26.32)** | 56 | 143 | 127 | 326 |
| **Cluster Analysis** | 29 | 69 | 62 | 160 |
| **H-bond Analysis (Cys83)** | 24 | 42 | 43 | 109 |
| **Commercially Available** | 12 | 38 | 41 | 91 |

**Table S2.** Molecular interactions between the Cdk5/p25 and ligands.

| Compound | Hydrogen bonds (≤ 3.5 Å) | | | | Other Interactions |
|---|---|---|---|---|---|
| | Amino Acid | Amino Acid Atom | Ligand Atom | Distance (Å) | |
| **Ref** | Cys83<br>Cys83<br>Asp86 | HN<br>O<br>OD1 | N16<br>H45<br>H27 | 2.2<br>1.7<br>1.6 | Ile10, Phe80, Ala31, Val64, Leu133, Val18, Lys33,Cys83, Asp84, Phe82, Gly11 |
| **Hit1** | Cys83<br>Cys83<br>Asn144 | HN<br>O<br>HD22 | O13<br>H41<br>O26 | 1.91<br>1.81<br>2.62 | Ile10, Gly11, Glu12, Gly13, Val18, Ala31, Lys33, Phe80, Phe82, Asp84, Gln85, Asp86, Lys89, Gln130, Asn131, Leu133, Asn131 |
| **Hit2** | Cys83<br>Cys83<br>Asn144 | HN<br>O<br>HD22 | O11<br>H38<br>O28 | 2.28<br>1.68<br>2.50 | Ile0, Gly11, Glu12, Gly13, Val18, Ala31, Lys33, Phe80, Phe82, Asp84, Gln85, Asp86, Lys89, Leu133, Ala143 |
| **Hit3** | Cys83<br>Cys83 | HN<br>O | O13<br>H41 | 1.67<br>1.83 | Ile10, Glu12, Gly13, Thr14, Val18, Asn131, Asn144, Ala31, Lys33, Val64, Phe80, Phe82, Leu133, Lys33, Ala31, Ala143 |
| **Hit4** | Cys83<br>Cys83<br>Asn144 | HN<br>O<br>HD22 | O10<br>H36<br>O26 | 1.80<br>1.84<br>2.08 | Ile10, Glu12, Gly13, Val18, Ala31, Lys33, Val64, Phe80, Glu81, Phe82, Asp84, Gln85, Asp86, Leu133 |

**Table S3.** IUPAC name, PubChem ID and Supplier information of the final hit compounds.

| Compounds | IUPAC* Name | PubChem ID | Vendor/Supplier (Product ID) |
|---|---|---|---|
| **Hit1** | (5S)-1-[3-(dimethylamino)propyl]-5-(4-ethoxy-3-methoxyphenyl)-4-[hydroxy-(4-methoxy-2-methylphenyl)methylidene]pyrrolidine-2,3-dione | 1050287 | ZINC (ZINC19929335) |
| **Hit2** | 1-[3-(dimethylamino)propyl]-4-[hydroxyl-(2-methyl-4-prop-2-enoxyphenyl)methylidene]-5-(4-methoxyphenyl)pyrrolidine-2,3-dione | 3146980 | Enamine (BG06474602) |
| **Hit3** | (5S)-5-(3,4-dimethoxyphenyl)-1-[3-(dimethylamino)propyl]-4-[(4-thoxy-3-methylphenyl)-hydroxymethylidene]pyrrolidine-2,3-dione | 1544486 | ZINC (ZINC19928667) |
| **Hit4** | 5-(3,4-dimethoxyphenyl)-1-[3-(dimethylamino)propyl]-4-[hydroxy-(4-methylphenyl)methylidene]pyrrolidine-2,3-dione | 2908872 | Enamine (BG06447444) |
